# The intriguing giant deer from the Bate cave (Crete): could paleohistological evidence question its taxonomy and nomenclature?

**DOI:** 10.1111/1749-4877.12533

**Published:** 2021-04-06

**Authors:** Maria Rita PALOMBO, Marco ZEDDA

**Affiliations:** ^1^ CNR‐IGAG c/o Department of Earth Sciences Sapienza University Roma Italy; ^2^ Department of Veterinary Medicine University of Sassari Italy

**Keywords:** *Candiacervus major*, Cretan deer, island rule, paleohistology, pituitary gigantism

## Abstract

The research describes for the first time a possible case of pituitary gigantism in fossil mammals, precisely in deer. The pathology was detected in 2 long bones (tibia and metatarsus) belonging to an individual of an unusual large size found at the Bate cave (Rethymnon, Northern Crete). It formed the basis of *Candiacervus major*, the largest among the endemic deer species recorded in the Pleistocene‐Early Holocene of Crete. Radiological and histomorphological examinations highlighted a reduction in cortical bone thickness and the presence of wide lacunae inside of the bone tissue. The pathological conditions suggest a pituitary gigantism diagnosis also supported by some morphological evidence, such as the extremely elongated distal part of the metatarsal diaphysis, the proportionally small proximal epiphysis, and some bone gracility. The diagnosis of a case of pituitary gigantism as presumed responsible for the extraordinary elongation of the tibia and the metatarsal bone is intriguing as they are, respectively, the paratype and the holotype of the *C. major*. The species represents a case of a deviation from the “island rule” in Pleistocene large mammals. The new evidence recommends a taxonomic and nomenclatural revision of this species. The main outcomes of this research are as follows: (i) a case of pituitary gigantism is described for the first time in an extinct mammal; (ii) it is underlined that paleohistology may provide interesting clues for disentangling taxonomic and nomenclatural issues; (iii) one of the very few cases of gigantism in insular mammals is being questioned.

## Graphical abstract

Morphological, radiological, and histomorphometric analyses conducted on the bones that are the paratype and holotype of *Candiacervus major* suggest that this giant Cretan deer would have been affected by pituitary gigantism. This implies that:

1) One case of pituitary gigantism is described in an extinct animal for the first time

2) A well‐known exception to the island rule ceases to exist

3) Paleohistology can make a contribution to solving taxonomic problems

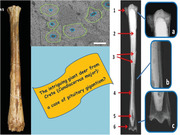

## INTRODUCTION

During the last couple of decades, studies in the fields of paleohistology and paleopathology have been increasing in relevance given their importance for better understanding the life history, paleoecology, behavior, paleohabitat, traumatology, and diseases in extinct and extant vertebrate species (see among several others, Castanet 2006; Waldron 2008; Canoville & Laurin 2010; Stein 2010; García‐Martínez *et al*. 2011; Jordana & Köhler 2011; Jordana *et al*. 2011; Thomas 2012; Marín‐Moratalla *et al*. 2013; Stein & Werner 2013; Amson *et al*. 2014; Martínez‐Maza *et al*. 2014; Kolb *et al*. 2015a,b; Bartosiewicz 2016, 2018; Lyras *et al*. 2016, 2019; Palombo & Zedda 2016; Olivier *et al*. 2017; Veitschegger 2017; Orlandi‐Oliveras *et al*. 2018; Veitschegger *et al*. 2018; Zoboli *et al*. 2018; Labarca & Pacheco 2019; Miszkiewicz *et al*. 2019; Woolley *et al*. 2019; de Souza Barbosa *et al*. 2020; Xing *et al*. 2020; Zedda *et al*. 2020). Interesting results about the growth rate and the seasonality of death are also achieved by the histological approach (Castanet *et al*. 2004; Sander & Andrassy 2006; Köhler & Moyà‐Solà 2009; Köhler *et al*. 2012; Kolb *et al*. 2015a).

Several histological studies of long bone tissue in vertebrates deal with the growth dynamics (Waskow *et al*. 2014; Kolb *et al*. 2015b; Veitschegger *et al*. 2018; Lyras *et al*. 2019). However, no histological analyses have been performed to date to scrutinize whether the particular large size of some extinct mammals may or may not be related to the pituitary gland activity.

This research aims to scrutinize the reliability of 2 alternative hypotheses: (i) the extraordinary large size shown by some bones of an endemic deer (*Candiacervus major*) found in the Late Pleistocene deposit of the Bate cave (Rethymnon, Crete) depends on an unusual increase in size due to the adaptive radiation process characterizing the evolution of the endemic Cretan deer (de Vos 2000, 2006); the excessive large size depends on pathological conditions. To investigate the consistency of the latter hypothesis, we analyzed the microstructure and the histological features of the bone tissue in the tibia and the metatarsal bone, which are respectively the paratype and the holotype of the *Candiacervus major* species (Capasso Barbato & Petronio 1986).

### Pituitary gigantism

The phenomenon of an excessive and prolonged growth due to chronic overactivity of the pituitary gland is a rare syndrome, sporadically recorded in nature and not described in depth in ancient remains. The cases reported to date in ancient historical time are very few. To date, only 3 human individuals show bones denoting an anomalous growth due to gigantism. One was found in a necropolis of Giza (Egypt), dated back to the Fifth Dynasty (Mulhern 2005; Galassi *et al*. 2017), the second in an Imperial Roman necropolis (3^rd^ century) near Rome (Italy) (Minozzi *et al*. 2012, 2015), and the third in a necropolis near to Cordoba (Spain), dated back to the VIII–XII centuries (Viciano *et al*. 2015).

It is well known (Kronenberg 2003; Rossellò‐Diez & Joyner 2015; Tritos & Klibanski 2016) that under normal physiological conditions, the growth in length of long bones at a young age is due to the stimulation of chondroblast cells in the metaphyseal cartilage by the growth hormone (GH) secreted by the pituitary gland. During the growing period, in case of GH hypersecretion (frequently caused by a benign pituitary adenoma), an over stimulation of the growth plates occurs, leading to an excessive bone lengthening (Mazziotti *et al*. 2018, 2019). Such endocrine disorder is named pituitary gigantism that produces an excessive increase in stature (Eugster & Pescovitz 1999; Schmidt *et al*. 2007). If the GH hypersecretion conversely occurs in adulthood, the bones change their shape due to the excessive periosteal apposition of bone tissue. This condition is named acromegaly (Scacchi & Cavagnini 2006; Chanson & Salenave 2008). Sometimes, the GH hypersecretion continues from a young age to adulthood so that the morphological traits due to gigantism and acromegaly are both present (de Herder 2009; Melmed 2009).

Although the macroscopic features of long bones (e.g. marked slenderness and fragility) have been frequently described in humans affected by gigantism (Mazziotti *et al*. 2018, 2019), less attention has been devoted to the microscopic characteristics of the bone tissue and its modifications. Evidence demonstrated that the GH excess may induce bone resorption resulting in a reduction of trabecular bone volume, trabecular thickness, and leads to bone tissue porosity (Ueland *et al*. 2002; Ueland 2005; Dalle Carbonare *et al*. 2018; Mazziotti *et al*. 2019). These histomorphological alterations are caused by the stimulation of osteoclast activity, leading to bone demineralization and can increase the risk of fractures related to bone fragility (Andreassen & Hoxlund 2001; Kužma *et al*. 2019). In sum, the characteristic features of long bones affected by this endocrine disorder mainly depend on the imbalance between the overstimulation of growth plates, leading to a progressive increase in bone tissue formation at the level of the metaphyseal zone, and increase in bone resorption in at the level of the epiphyseal and diaphyseal zones. As a result, the long bones are slender, thin, and fragile.

### Cretan Late Pleistocene fauna and its deer

During the Late Pleistocene, the island of Crete was inhabited by an ecologically unbalanced mammalian fauna (*Mus catreus* and *Mus minotaurus* faunal complexes) dominated by endemic deer and including a dwarf elephant *Palaeoloxodon creutzburgi*, an otter *Lutrogale cretensis*, a shrew *Crocidura zimmermanni*, and a giant mice *Mus minotaurus* (e.g., Lax 1996; Palombo 2009; Van der Geer *et al*. 2010 with bibliography).

For decades, issues related to the systematics, taxonomy, and evolutionary patterns of the Cretan deer have been attracting the attention of researches who extensively analyzed also their differences in size, feeding behavior, and locomotion. The Cretan deer phylogenetic relationships remain, however, a matter of major debate (Kotsakis *et al*. 1976; de Vos 1979, 1996, 2000, 2006; Capasso Barbato & Petronio 1986; Capasso Barbato 1989, 1990, 1992, 1995; Caloi & Palombo 1995, 1996; Van der Geer *et al*. 2006, 2013; Iliopoulos *et al*. 2010; Van der Geer 2014, 2018; Altamura 2016).

Some authors, for instance, considered the small Cretan deer as a monophyletic group phylogenetically related to Megacerini (e.g., Sondaar & Boekschoten 1967; Kurtèn 1968; Malatesta 1980) or to a still unidentified ancestor (de Vos 1996, 2000, 2006). Others hypothesized that the Cretan deer originated from 2 dispersal phases (e.g., de Vos 1979, 1984; de Vos & Dermitzakis 1986; Caloi & Palombo 1996). Others (e.g., Capasso Barbato 1990) believed that the Cretan deer is a polyphyletic group, stemming from 2 different continental deer lineages (*Praemegaceros* and *Cervus)*.

Authors also disagree about the number of deer species present on Crete. de Vos, for instance, firstly distinguished 6 monospecific size‐groups (de Vos 1979), based on the limb bone biometrical data from the localities known at that time. Later de Vos (1984) split the size‐group 2 in 3 species. Consequently, the author considered present on Crete at least 8 taxonomic units (*Candiacervus ropalophorus*, *Candiacervus* sp. IIa, IIb, IIc, *Candiacervus cretensis*, *Candiacervus rethymnensis*, *Candiacervus* sp. V, *Candiacervus* sp. VI), based on the skull morphology and proportion (4 morphotypes of skull, 5 types of antlers, 3 morphotypes of P^2^, and 3 size groups in the teeth), as well on long bone length.

Conversely, Capasso Barbato (1989, 1990, 1992) recognized on Crete 5 *Cervus* endemic species and 2 subgenus. She ascribed to the new subgenus *Leptocervus* the species *Cervus (Leptocervus) rethymnensis*, *Cervus (Leptocervus) dorothensis*, and *Cervus (Leptocervus) major*. Accordingly, the Cretan deer would constitute a polyphyletic group, whose ancestors entered Crete at different times.

To date, the debate is still open even if the majority of researchers consider the Cretan deer as a monophyletic group (including not less than 5 and not more than 8 species, belonging to 6 size‐groups [see below]), stemming from a single continental, still debated ancestor. It probably reached the island by swimming during a Middle Pleistocene glacio‐eustatic low‐sea‐level stand (e.g. de Vos 1996, 2000, 2006; van der Geer *et al*. 2010, 2013; van der Geer 2018; Palombo 2018; Heckeberg 2020).

When the mainland ancestor of the Cretan deer arrived on the island, many habitats and empty niches were likely available due the absence of other middle‐sized herbivores, deer competitors on the mainland (Palombo 2009, p. 349, Fig. 7). This fact allowed deer to undergo an adaptive radiation, typically documented by the presence of taxa of different size in the same area at the same time (e.g. de Vos 2000, 2006).

The genus *Candiacervus* includes several coexisting species, each showing not only different size but also peculiar cranial, dental, and long bone morphological traits, suggesting they were adapted to different habitats and occupied a diverse set of niches (see e.g. de Vos 1984, 1996; Caloi & Palombo 1995, 1996; Palombo 2009; Altamura 2016; van der Geer 2018 and references therein).

**Figure 1:**
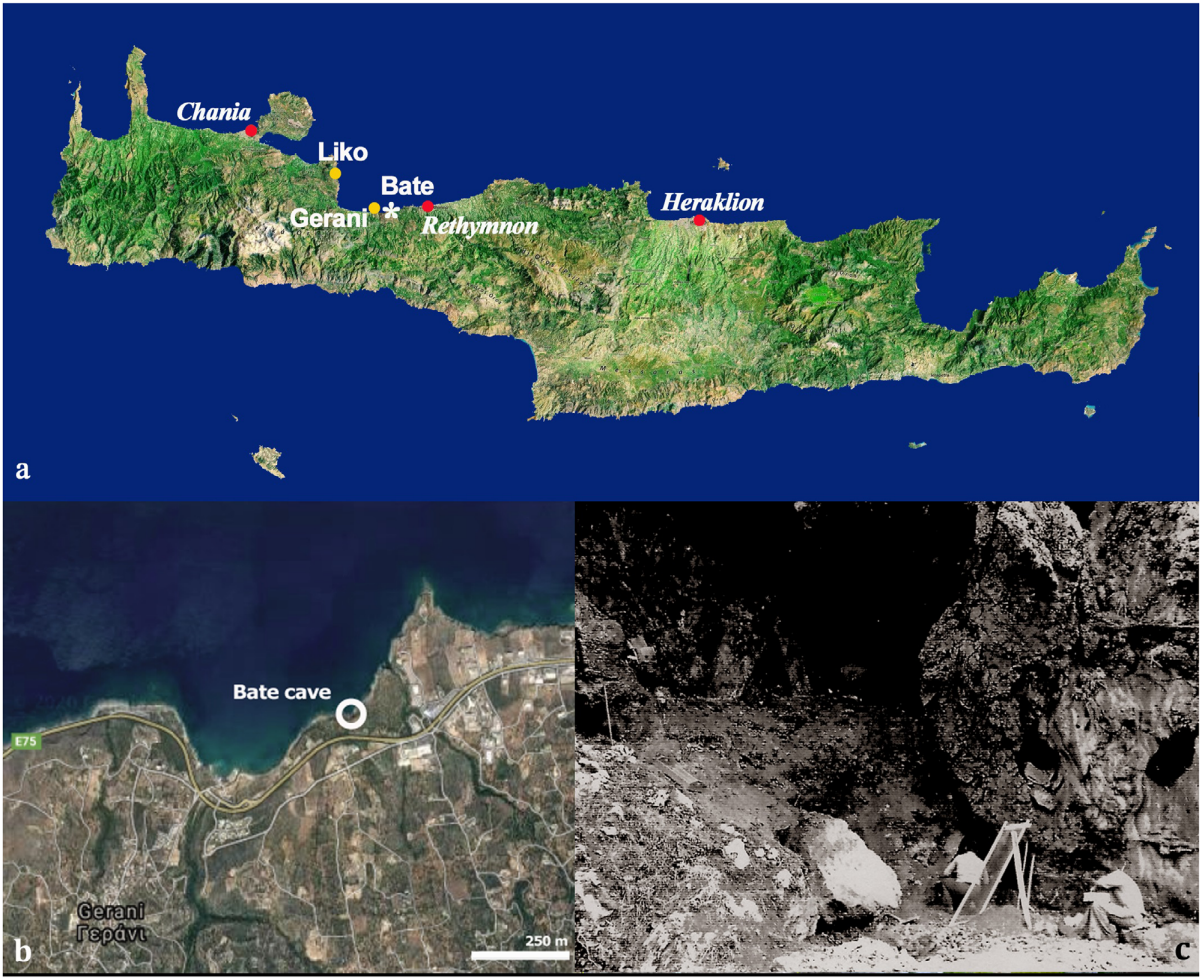
Bate cave (Rethymnon): (a,b) localization of the Bate cave within the isle of Crete; (c) Bate cave in 1975 during a geological survey made by a team of the Sapienza University of Rome (Italy).

The taxa of small body size (deer of the size group 1 and 2 of de Vos (1979, 1984), recently ascribed to 4 species, *Candiacervus ropalophorous* de Vos, 1984, *Candiacervus devosi* van der Geer, 2018, *Candiacervus listeri* van der Geer, 2018, *Candiacervus reumeri* van der Geer, 2018 (van der Geer 2018)) are recorded by a plentiful fossil record in several localities, mainly caves, opening along the island northern coastline (Iliopoulos *et al*. 2010). The 2 moderately larger species (size groups 3, *Candiacervus cretensis* (Simonelli, 1907), and 4, *Candiacervus rethymnensis* Kuss, 1975 (de Vos 1979, 1984)) are also present in some localities with a number of specimens. Conversely, only a few remains of the large species *Candiacervus dorothensis* (Capasso Barbato, 1992) (size groups 5 of de Vos (1979, 1984)) are reported from the Bate cave, while a small number of bones and fragments of the largest deer *Candiacervus major* (Capasso Barbato & Petronio, 1986) (size groups 6 of de Vos (1979, 1984)) were found at the Bate and Liko cave (Fig. 1). The latter species, which at the Bate cave is represented by a few remains belonging to a single individual, deserves particular interest.

### Bate cave and its faunal record

The Bate cave, located on the Northern Cretan coast, not far from the Zourida gorge, in the cliffs below the Rethymnon‐Chania National Road (Fig. 1), was discovered in 1975 during a geological survey made by a team of the Sapienza University of Rome (Italy) led by Alberto Malatesta (Kotsakis *et al*. 1976). The fossil assemblage of the Bate cave belongs to the youngest Pleistocene Cretan fauna complex, the so‐called *Mus minotaurus* “sub‐zone” (Mayhew 1977). The age of the fossiliferous deposit is not firmly established because of the inconsistency of data obtained by different authors/methodologies (see Lax 1996). Belluomini and Delitala (1983, 1988) indicated an age from 63 500 ± 20% to 69 000 ± 20% years (Amino Acid Racemization, AAR, dating on deer bones). Reese *et al*. (1996) warned that AAR dating done on bones are “very much less reliable than tooth enamel.” The authors’ AAR dating on deer teeth are indeed significantly older: 152 000 ± 20% to 105 000 ± 20%, suggesting a late Middle to early Late Pleistocene age for the deposits of the Bate cave (Rees *et al*. 1996).

The vertebrate fauna retrieved from poorly stratified fossiliferous layers filling the Bate cave includes, among others, a rich sample of deer, belonging to 5 size‐groups (size‐group 1, 2, 3, 5, and 6 of de Vos 1979). The record of the newly created species *Candiacervus major* (size‐group 6) counts few bones (6 vertebrae, including the epistropheus, and the third and fourth cervical vertebrae, 2 incomplete humeri, 1 radius‐ulna, 3 pelvis fragments, 1 incomplete femur, 1 complete tibia, 1 complete metatarsus and 2 fragments, 1 phalanx). The complete/nearly complete bones likely belonging to a single individual as suggested by their dimensions (cfr. Capasso Barbato & Petronio 1986). According to the diagnosis provided by Capasso Barbato and Petronio (1986), the species is characterized by very long and slender limb bones.

## MATERIALS AND METHODS

### Materials

The *Candiacervus major* tibia (specimen number 28 in Capasso Barbato & Petronio 1986) and metatarsal bone (specimen number 30 in Capasso Barbato & Petronio 1986) herein studied are part of the rich fossil sample collected in the 70s at the Bate cave and currently stored in the University Museum of Earth Science (MUST, previously Museum of Paleontology), Department of Earth Sciences of the Sapienza University (Rome, Italy).

We compared the dimensions and proportions of the *C. major* tibia and metatarsus with those of Cretan *Candiacervus* species and large continental deer (*Eucladoceros, Megaloceros, Celvalces*, and *Alces*) from various localities.

The histomorphological features shown by the bones of this species have been compared with those of a left metatarsus (MPUR/V coll. Bate, n 16b in Capasso Barbato 1989) belonging to the group of the smallest deer found in the cave. The left metatarsal bone could be identified as *C. ropalophorus* (and so called here from now on), though the hypothesis that it could represent an individual belonging to one or another of the species of size‐group 2 (*Candiacervus* sp. II, de Vos 1979, 1984) cannot be excluded.

### Methods

Bone measurements were taken using a digital caliper accurate to 0.01 mm. Multivariate statistical analysis have been performed by means of PAST software (Hammer *et al*. 2001).

The bones were submitted to radiological analysis. The digital radiographic images of the tibia and metatarsus were captured exposing the bones at a distance of 120 cm from the X‐ray tube programmed with 100 kV, 10 mA, 5 s of time. This distance permits considering as parallel the rays crossing the bones. The radiological images corresponding to the central part of the shaft enable us to measure the dimension of the medullary cavity and the thickness of the cortical bone (Croker *et al*. 2016). The parameter *R*/*t* (the ratio between the radius of the whole bone section and the cortical thickness) has been calculated in a tibia and metatarsus of *C. major* and *Eucladoceros giulii* from Untersmassfeld (Germany) (tibia num. IQW 1980/16 501 (Mei. 16 022) metatarsus num. IQW 1980/16 500 (Mei. 16 021); Senckenberg Research Station of Quaternary Palaeontology Weimar, collection) following Currey and Alexander (1985). The obtained parameters have been compared to the Cervidae *R*/*t* parameters available in literature (Laurin *et al*. 2011; Amson & Kolb 2016).

The radiotransparency of the bone tissue was estimated by subjecting to a colorimetric analysis a square of 3 mm per side of the diaphysis radiological images, using the Scion image software (Scion Corporation, Frederick, MD, USA). The reference values were 0 (white, corresponding to maximum radiopacity) and 255 (black, corresponding to maximum radiotransparency) (Mura *et al*. 2013). A metatarsus of an extant *Cervus elaphus* was used as reference comparison (mean value 78).

A sample approximately 0.5 cm thick was removed from the tibia and metatarsal bone at the level of the diaphysis by means of a steel cutting disk mounted in a mini‐portable electrical screwdriver (Bosch). The small bone blocks were embedded with a bicomponent epoxy resin (Araldite 2020, Huntsman, Basel, Switzerland), using a degassing vacuum chamber (Ablaze1, Ablaze, Hong Kong) to avoid bubbles. It was cut and ground by means of a thin section cutting and grinding machine (Geoform102, Metkon Instruments, Bursa, Turkey) and an abrasive cutting machine (Metacut302, Metkon Instruments) by using a diamond disk (Dimos Ø 250, Metkon Instruments). Sections with final thickness of 80 μm were mounted onto glass slides with Eukitt (Merck, Darmstadt, Germany) and covered. Sections were observed and photographed using a Zeiss Axiophot microscope at 2.5×, 10×, and 20× magnifications under transmitted light and polarized light. Images were digitalized and the measurements have been taken using the Scion Image software. The bone tissue types have been described following the classification proposed by Francillon‐Vieillot *et al*. (1990). The same protocol and histomorphometric procedures were followed in order to compare morphological and histomorphological features of the *C. ropalophorus* metatarsal bone (Palombo & Zedda 2016).

The analyzed samples and the slides are deposited in the paleontological slide collection (PSC) at the Section of Anatomy of the Department of Veterinary Medicine of the University of Sassari (Italy) and are available for repeatability. The slide catalog numbers are the following: *C. major* tibia from PSC1844 to PSC1859, metatarsus from PSC1860 to PSC1868; *C. ropalophorus* metatarsus from PSC1869 to PSC1875.

## RESULTS

### Main morphological features and biometrical peculiarities

The tibia and metatarsus of *C. major* belong to an adult individual. The epiphyses and the diaphysis are fully fused as a single ossified structure, so that the growth plate cartilage (or “metaphysis”) is completely ossified (see Calderòn *et al*. 2019 as regards to the fusion time in deer).

**Figure 2:**
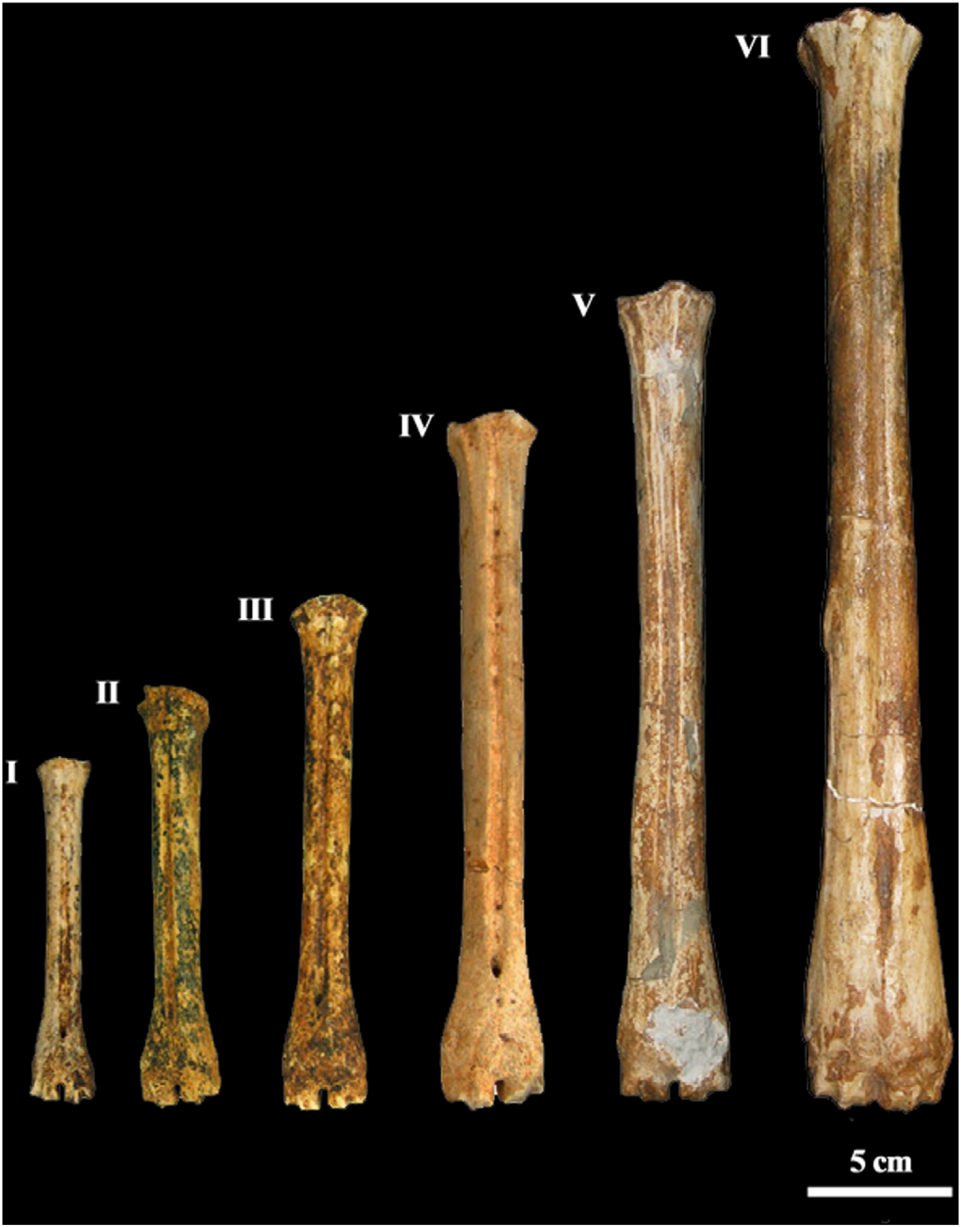
Metapodial bones of the Cretan endemic deer numbered (from the left to the right) according to the size‐groups defined by de Vos (1979, 1984): *Candiacervus ropalophorous* de Vos 1984 (=size‐group I), *Candiacervus* spp. II (=size‐group II), *Candiacervus cretensis* (Simonelli 1907) (=size‐group III), *Candiacervus rethymnensis* Kuss 1975 (=size‐group IV), *Candiacervus dorothensis* (=size‐group V) (Capasso Barbato 1992), and the metatarsus from Bate cave, holotype of the species *Candiacervus “major”* (=size‐group VI) (Capasso Barbato & Petronio 1986).

The main features of both long bones are their remarkable length and the extreme gracility of the medio‐distal part of the tibia and the medio‐proximal part of the metatarsus. In particular, the metatarsus has a narrow proximal epiphysis, a massive and progressively enlarged distal portion of the diaphysis (with a high positioned foramen), and a very short distal epiphysis (Fig. 2, Table 1) (for further details refer to Capasso Barbato & Petronio 1986, comparison tables, pp. 72–73, 76–77). The *C. major* metatarsal length is about 334%, 293%, 225%, 168%, and 134% than those of, respectively, *C. ropalophoros*, *Candiacervus* spp.II, *C. cretensis*, *C. rethymnensis*, and *C. dorothensis* (Table 2). The estimation of the shoulder height, based on metapodial length (Godynicky 1965), confirms high stature of *C. major* (Table 3), which was about 3.4, 2.9, 2.6, 1.8 and 1.3 times taller than *C. ropalophoros*, *Candiacervus* spp.II, *C. cretensis*, *C. rethymnensis*, and *C. dorothensis* (size‐group 5), respectively.

**Table 1:** Measurements of tibia and metatarsus of *Candiacervus major*

	Tibia	Metatarsus	
Measurements (mm)	Bate cave	Bate cave	Liko^†^
Total length	483.6	406.2	—
Mediolateral width of the proximal epiphysis	80.2	45.9	—
Antero‐posterior depth of the proximal epiphysis	79.8	46.5	—
Mediolateral width of the diaphysis	28.5	27.1	—
Mediolateral width of the distal diaphysis portion	50.3	—	—
Mediolateral width of the distal epiphysis	48.8	46.2	50.6
Antero‐posterior depth of the distal epiphysis	30.7	31.9	33.6

^†^ Data from de Vos (1979).

**Table 2:** Comparison among the length of the tibia and metatarsus of all Cretan *Candiacervus* species

	Tibia			Metatarsus		
Taxa	Min–Max	M	*N*	Min–Max	M	*N*
*Candiacervus major*	483.6	483.6	1	406.2	406.2	1
*Candiacervus* from Gerani IV^†^ (size‐group 1)	145.3–175.5	161.5	68	110.0–131.0	121.64	66
*Candiacervus* from Liko^†^ (size‐group 2)	169.9–205.2	186.5	21	123.2–152.5	138.4	45
*Candiacervus* size‐group 3^†∧^	—	—	—	166.0–180.0	174.0	3
*Candiacervus* size‐group 4^†^	—	—	—	217.5–241.8	229.65	2
*Candiacervus cretensis* ^∧^	248.0	248.0	1	—	—	—
*Candiacervus cretensis* ^§^	—	—	—	110.0–145.0	—	—
*Candiacervus cretensis*°	—	—	—	123.0–132.0	128.4	5
*Candiacervus* size‐group 4^†^	313.6–314.9	314.25	2	217.5–241.8	229.65	2
*Candiacervus ropalophorus*/*Candiacervus* spp.II^∧^	148.3–201.0	171.42	75	99.0–148.5	127.54	128
(*Candiacervus ropalophorus*)^∧∧^	148.3–169.0	163.4	33	99.0–123.8	114.70	32
(*Candiacervus ropalophorus* vel *C*. spp.II)^∧∧^	—	—	—	124.0–131.0	129.51	55
(*Candiacervus* spp.II)^∧∧^	170.0–201.0	177.72	42	131.5–148.5	138.29	42
*Candiacervus dorothensis* ^∧^	370.0	370.0	1	301.0–301.4	301.2	2

Measurements (in mm) from: ^†^de Vos (1979), ^§^Kuss (1975), °Malatesta (1980), ^∧^Capasso Barbato (1989) (= *C. ropalophorus*), ^∧∧^tentative separation of the specimens identified as *Candiacervus ropalophorus* by Capasso Barbato (1995) in 2 groups (*C. ropalophorus* = size‐group 1 and *Candiacervus* spp.II = size‐group 2) according to the dimensional range given by de Vos (1979) for metatarsal bones. M, mean value; *N*, number of bones.

**Table 3:** Comparison among the shoulder height of all Cretan deer of the genus *Candiacervus* on the basis of the index of Godynicky (1965)

	Shoulder height		
Taxa	Min–Max	M	*N*
*Candiacervus major*	—	1848	1
*Candiacervus* size‐group 1^†^	501–596	553	66
*Candiacervus* size‐group 2^†^	561–594	630	45
*Candiacervus* size‐group 3^†∧^	755–819	792	3
*Candiacervus* size‐group 4^†^	990–1100	1045	2
*Candiacervus cretensis* ^§^	501–660	—	—
*Candiacervus cretensis*°	560–601	584	5
*Candiacervus ropalophorus/Candiacervus* spp. II^∧^	450–676	580	128
(?*Candiacervus ropalophorus*)^∧∧^	450–589	552	70
(?*Candiacervus* spp. II)^∧∧^	592–676	614	58
*Candiacervus dorothensis* ^∧^	1370–1371	1370	2

Values (in mm) calculated from length of long bones reported by ^†^de Vos (1979), ^§^Kuss (1975), °Malatesta (1980), ^∧^Capasso Barbato (1989), ^∧∧^Capasso Barbato (1995).

**Figure 3:**
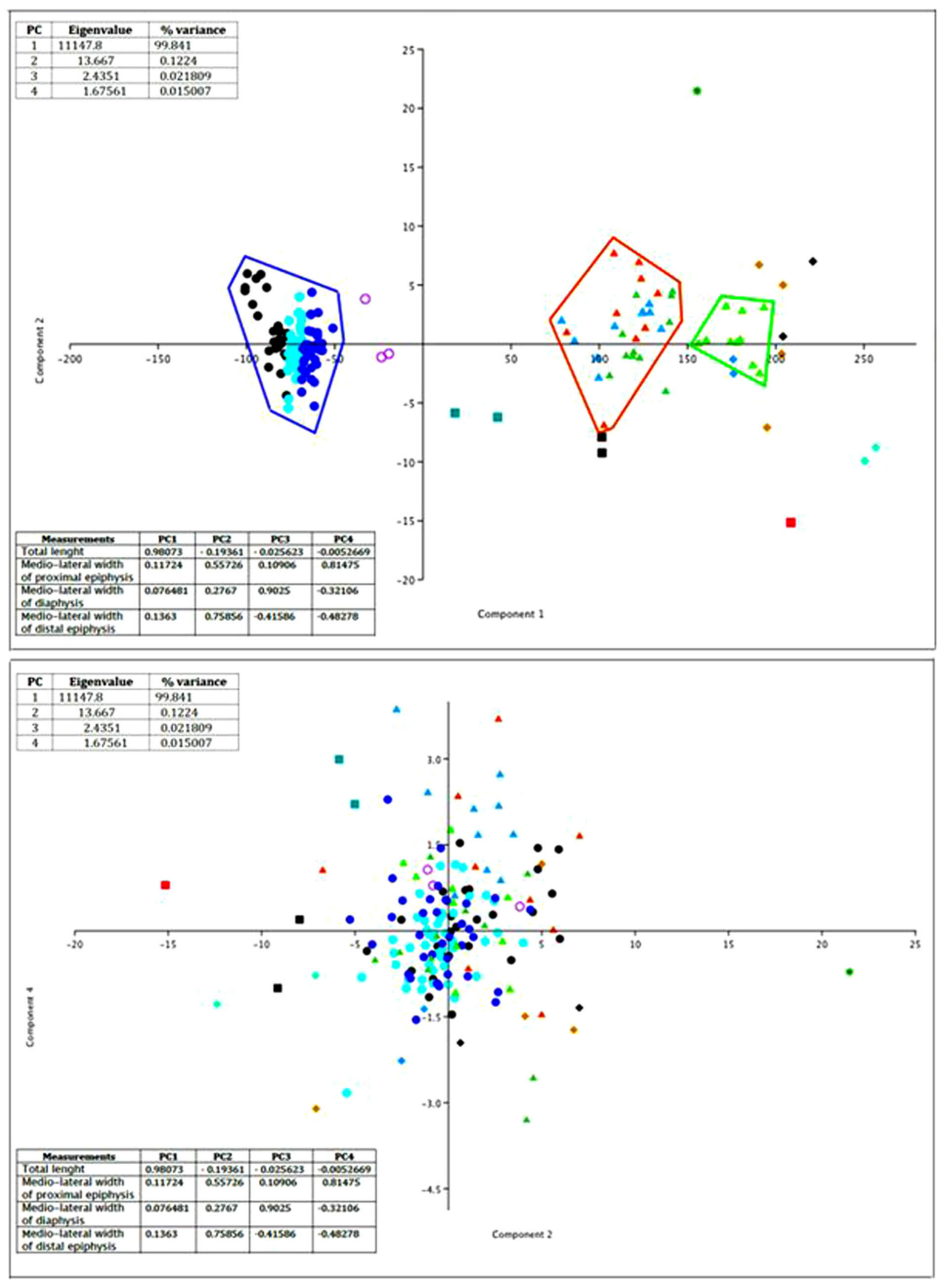
Diagrams resulting from the principal components analysis (PCA) computed by using as variables the total length and the mediolateral width at the proximal and distal epiphysis and diaphysis of metatarsal bones of Cretan deer and slender large continental deer (cases). Above: PCA graph based on the first component, in which the total length is the most loading variable, and the second component, in which the mediolateral width of the proximal epiphysis is the most loading variable. Below: PCA graph based on the second component and the fourth component, in which the mediolateral width of the distal epiphysis is the most loading variable. Symbols—*Candiacervus*: Black dots, *C. ropalophorus* from Bate cave (1); blue dots, *C*. spp.2 Bate (1); aqua dots, *C. ropalophorus/C*. spp.2 from Bate; black filled squares, *C. dorothensis* Bate (1); red filled square, *C. major* from Bate cave; violet circles, *C. cretensis* from Liko (2); dark cyan filled squares, *C. rethymnensis* from Liko and Mavro Mouri (2). *Eucladoceros*: Light green filled triangles, *E. giuli* from Untermassfeld (Germany) (3); light brown filled triangles, *E. dicranios* from Olivola (Italy); orange filled triangles, *E. dicranios* vel *E. ctenoides* from Vardarno (Italy); dark green filled triangles, *E. ctenoides* ( = *E. senezensis vireti*) from Saint Vallier (France) (6); doger blue filled triangles, *E. ctenoides* ( = *E. senezensis senezensis*) from Senèze France (6). *Megaceros*: dark green star, *M. giganteus* from Villereversure (France) (6). *Cervalces* : azur filled diamonds, *Cervalces gallicus* from Senèze (France) (6); aquamarine filled diamonds, *Cervalces latifrons* from Tiraspol (Moldova) and Tunguska river (Siberia) (5). *Alces alces*: black filled diamonds, *A. alces* from North America (Alaska, Isle Royale, and other mainland US countries) (average value) (4); light brown, *A. alces* from Norge (6). (1) Capasso Barbato (1989); (2) Kuss (1975); (3) Kahlke (1997); (4) A. Valli unpublished; (5) Sher (1971); (6) Silva *et al*. (2014).

The peculiar proportions of the *C. major* metatarsus are highlighted by the results of Principal Component Analysis (PCA) (Fig. 3). In the PCA graph, the metatarsus sets apart because the proximal and distal epiphysis are, respectively, mediolaterally narrower and larger than in other mainland and continental deer, even similar in size to *C. major*.

The slenderness index (SI, the ratio percentage of the minimum breadth, measured near the middle of the shaft and the maximum length), of the tibia (SIt = 0.066) and the metatarsal bone (SIm = 0.073), denotes the gracility of the diaphysis in *C. major* metatarsus with respect to other Cretan deer from Bate cave (*C. ropalophorous*, *C. ropalophorous* vel *C*. spp.2, *Candiacervus* spp. II, *C. dorothensis*), *C. cretensis* from Liko, *C. rethymnensis* from Liko and Mavro Mouri, and slender large continental deer such as Epivillafranchian *Eucladoceros giulii* from Untermassfeld (Germany), Villafranchian *Eucladoceros dicranios* vel *E. ctenoides* from Italy, Villafranchian *Eucladoceros ctenoides* (*=E. senezensis*) from France, *Cervalces gallicus* from Senèze (SIm = 0.082, 0.085), *Cervalces latifrons* from Moldova and Siberia), and extant *Alces alces* from Norge and North America) (Table 4; source of data and references as in Fig. 3).

**Table 4:** Comparison among the slenderness index (the ratio percentage of the minimum breadth, measured near the middle of the shaft and the maximum length) in the metatarsal bones of *Candiacervus major*, the other Cretan *Candiacervus* species, and extinct and extant continental large deer (data source and references as in Fig. 3)

Metatarsus	
Taxa	Slenderness index (SIm)
*Candiacervus major* (Bate cave)	0.073
*Candiacervus ropalophorous* (Bate cave)	0.086–0.141; M 0.134
*Candiacervus ropalophorous* vel *C*. spp. II (Bate cave)	0.084–0.119; M 0.101
*Candiacervus* spp. II (Bate cave)	0.083–0.124; M 0.101
*Candiacervus cretensis* (Liko cave)	0.097–0.104; M 0.098
*Candiacervus rethymnensis* (Liko cave)	0.084
*Candiacervus dorothensis* (Bate cave)	0.079, 0.078
*Eucladoceros dicranios* vel *E. ctenoides* (Italy)	0.078–0.104; M 0.092
*Eucladoceros ctenoides* (France)	0.078–0.11; M 0.090
*Eucladoceros giulii* (Untermassfeld, Germany)	0.079–0.089; M 0.083
*Cervalces gallicus* (Senèze, France)	0.082, 0.085
*Cervalces latifrons* (Moldova)	0.1
*Cervalces latifrons* (Siberia, Russia)	0.084
*Alces alces* (Norge)	0.079–0.089; M 0.084
*Alces alces* (North America)	0.080–0.085, M 0.083

### Radiology

**Figure 4:**
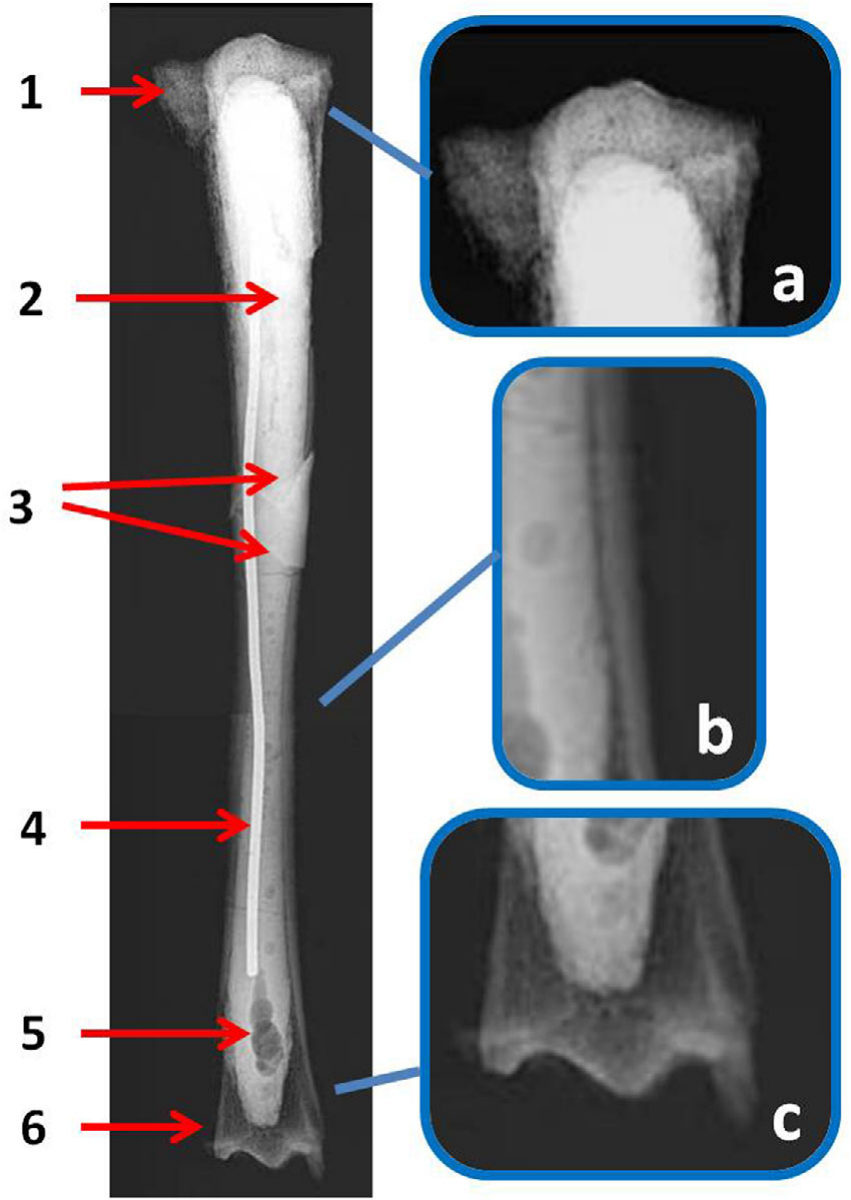
*Candiacervus major*, Bate cave (Rethymnon), tibia (specimen number 28 in Capasso Barbato & Petronio 1986). Radiological analysis in craniocaudal view. Wide bone reabsorption area in the proximal (1) and distal (6) epiphyses. The entire medullary cavity has been filled with a sealing material (2) showing air bubbles (5) and incorporating a metal pin (4) in order to strengthen the bone. This suffered a *post mortem* fracture whose breaking lines can be seen (3). On the right, some enlarged details of the epiphyses (a, c) and diaphysis wall (b) show the rarefied status of the bone tissue.

**Figure 5:**
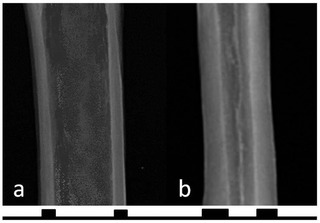
Radiological comparison of the central part of the metatarsal diaphysis between *Candiacervus major* (a) and *Candiacervus ropalophorus* (b). A reduction in cortical bone thickness in the diaphysis leads to a remarkable enlargement of the medullary cavity in *C. major*.

The radiological analysis of the tibia and metatarsus of *C. major* from the Bate cave confirms the achievement of adulthood due to the absence of the metaphyseal lines and shows a remarkable enlargement of the medullary cavity due to the thickness reduction of the cortical bone along the whole diaphysis (Fig. 4). The cortical bone tissue at level of mid‐diaphysis (measured in the mediolateral radiographic view) is 4.7 mm thick in the tibia and 4.5 mm thick in the metatarsus. At the same level, the diameter of the medullary cavity is 19.1 mm in the tibia and 18.1 mm in the metatarsus, with *R*/*t* of 3.10 and 3.04, respectively (Table 5). The diaphyseal bone tissue is proportionally thicker (3.9 mm; *R*/*t* = 1.67) in the metatarsus of *C. ropalophorus* (Fig. 5).

**Table 5:** Comparison between morphometrical data of the tibia and metatarsus of *Candiacervus major* and *Eucladoceros giulii*

		Mediolateral width of the diaphysis	Radius of the whole section (*R*)	Medullary cavity width	Cortical bone thickness (*t*)	*R*/*t*
*Candiacervus major*	Tibia	28.5	14.25	19.10	4.7	3.10
	Metatarsus	27.1	13.55	18.10	4.5	3.04
*Eucladoceros giulii*	Tibia	40.6	20.3	20.1	10.25	1.98
	Metatarsus	29.2	14.6	15.1	7.05	2.07

The measurements of *C. major* are taken from the mediolateral view of the radiological images at level of the central part of the shaft and those of *E. giulii* from Fig. 10.

The radiotransparency of the diaphyseal and epiphyseal bone tissue is higher than in normal bone tissue, indicating a widespread bone porosity in both bones.

The radiotransparency values were 191 in the tibia epiphyses (mean), 185 in the tibia diaphysis, 188 in the metacarpus epiphyses (mean), and 179 in the metacarpus diaphysis. The high radiotransparency of *C. major* bones is confirmed by the mean value (184) that is more than twice the reference value of *C. elaphus*.

The radiological examination enables us to reconstruct some events that occurred to the tibia after its finding. Due to the bone fragility, the tibia broke in 2 pieces during the excavation and was restored. During the restoration works, not affecting the cortical compact tissue, the spongy tissue was partially removed. The cavity was filled with mastic and the bone was reinforced by introducing an iron cylinder in the medullary cavity (Fig. 4).

### Histomorphology

**Figure 6:**
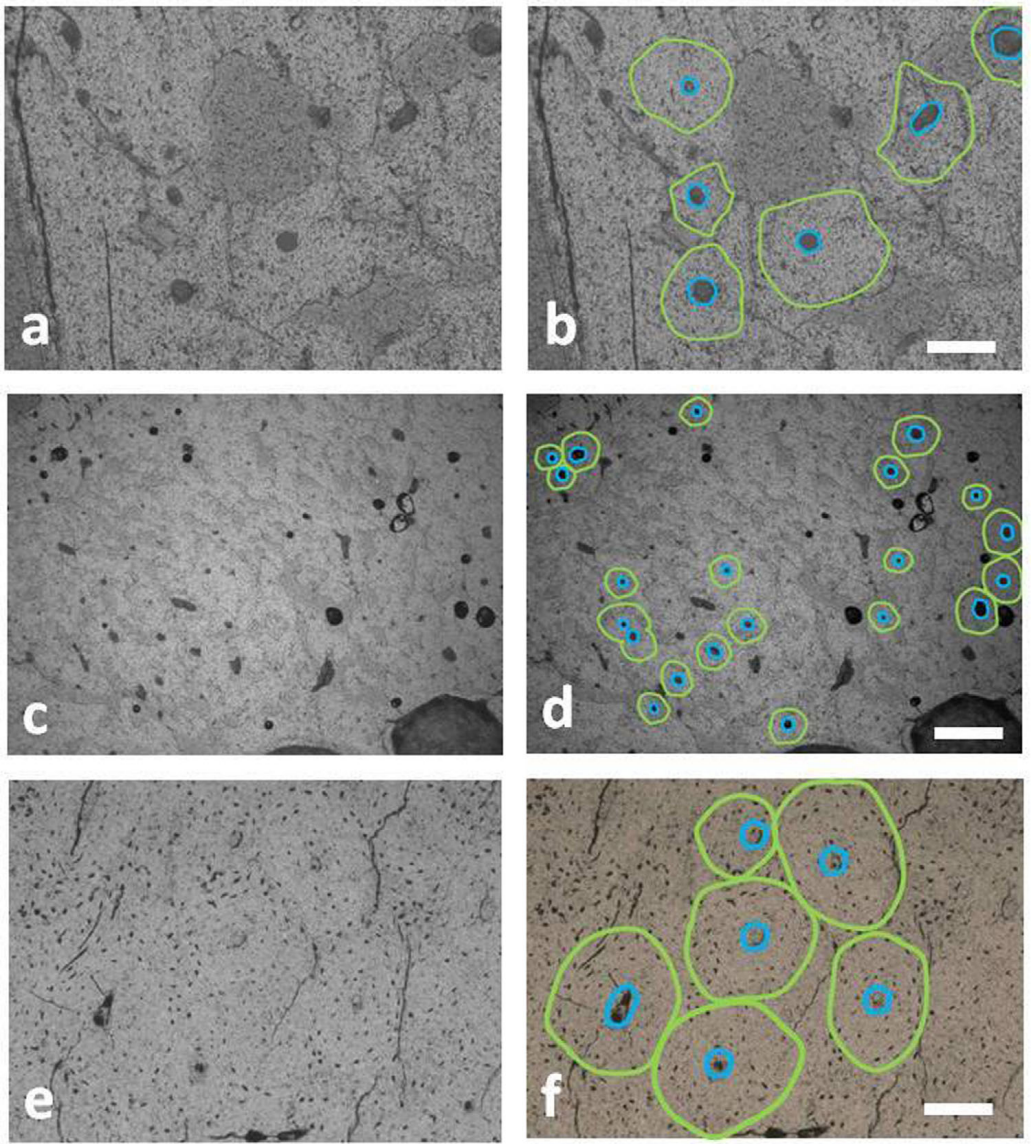
Secondary osteons in the diaphyseal bone of the tibia (a,b) and metatarsal bone (c,d) of *Candiacervus major* and metatarsal bone of *Candiacervus ropalophorus* (e,f). The green line identifies the bone lamella delimiting the osteon area and the blue line indicates the perimeter of the Haversian canal. Bar = 80 μm. (transmitted light microscope; (a) PSC 1848; (c) PSC1867; (e) PSC1873).

Light microscope observations reveal the presence of the plexiform, irregular Haversian tissue, and sometimes dense Haversian bone tissue. In the osteons, the external cement line is not always identifiable, but the position of the osteocyte lacunae, circularly arranged, indicates the presence of the bone lamellae surrounding the Haversian canal (Fig. 6). The use of a polarized light microscope confirmed this arrangement.

The mean diameter of the *C. major* osteons (obtained by measuring 43 secondary osteons) is 64 μm and that of Haversian canals is 18 μm. The osteons are almost circular as indicated by their very low eccentricity (*e* = 0.16). The tibial osteon size and their Haversian canals are 25% bigger than that of metatarsal ones. In the metatarsal bone of *C. ropalophorus*, the size of secondary osteons (*n* = 18) are 13% bigger (mean diameter 72 μm) than those of *C. major*, whereas the size of the Haversian canals is nearly the same. The secondary osteons of *C. ropalophorus* are moderately elliptical (*e* = 0.43).

**Figure 7:**
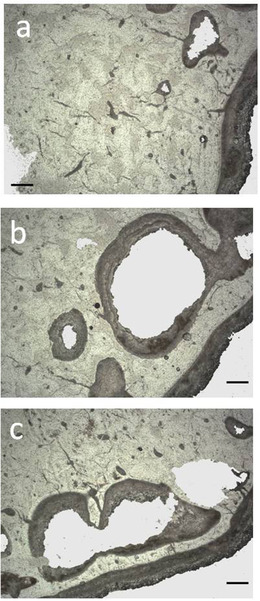
Histological pictures from the diaphysis of the *Candiacervus major* from Bate cave: tibia (a,b), metatarsus (c). Wide multiple cavities inside of bone wall can be noted in the subperiosteal zone (a,c) and subendosteal zone (b). Bar = 80 μm. (transmitted light microscope; (a) PSC1854; (b) PSC1857; (c) PSC1865).

One of the main peculiarities of the bone tissue of *C. major* is related to the presence of wide cavities inside of diaphyseal bone wall in both the tibia and metatarsus (Fig. 7). The empty spaces in the bone tissue are elongated parallelly to the periosteal or endosteal surfaces and seem often interconnected to each other. Their size can reach to a few hundred microns. A thin layer of dark, concreted sediment, sometimes more than 70 μm thick, covers the surface of these cavities. Sometimes, the dark sediment creeps into the cracks present in the bone wall. These wide cavities make the compact diaphyseal bone very porous and irregular. The area occupied by these empty spaces is about 18% of the diaphyseal bone wall. The bone tissue of the *C. ropalophorus* lacks cavities and looks compact and homogeneous.

### Skeletochronology and growth marks study

**Figure 8:**
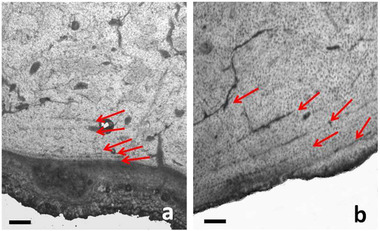
Lines of arrested growth (LAGs) in the tibia of *Candiacervus major* (a) and metatarsus of *Candiacervus ropalophorus* (b). The arrows indicate the last 5 subperiosteal lines suggesting an age of death more than 6 years. For skeletochronology considerations, see the text. Bar = 80 μm. (transmitted light microscope; (a) PSC1854; (b) PSC1871).

In the outer zone of the diaphyseal bone wall of *C. major* tibia and metatarsus, a few dark lines parallel to the periosteal surface are detectable somewhere in the histological slides. Following some studies dealing with the significance of these lines (Castanet *et al*. 2004; Köhler *et al*. 2012; Kolb 2016; Nacarino‐Meneses *et al*. 2016a,b; Calderòn *et al*. 2019), the lines could be regarded as lines of arrested growth (LAGs), which are often associated with ectothermic animals, but also present in large mammals such as some deer (Woodward Ballard *et al*. 2013). Their analysis permits inferring not only the approximate age of death but also the growth rate during the life of the analyzed individual. Each line corresponds to a period in which a low periosteal activity occurs, while the bone tissue between 2 lines, named growth zone, corresponds to the period with high activity. In the *C. major* tibia and metatarsus, at least 5 LAGs can be detected (Fig. 8), suggesting that the deer might be about 6 years old at death. In the tibia, the first 3 growth zones (from the deepest part to the periosteal surface) measure about 40 μm, while the last 2 growth zones only 15 μm. The estimated growth rate (i.e. the bone tissue formed each day in the growth period, Kolb 2016) is 0.16 μm/d during the 2^nd^, 3^rd^, and 4^th^ year of life—at the time that the first 3 growth zones formed—and of 0.05 μm/d during the 5^th^ and 6^th^ year of life, when the last 2 growth zones formed. The metatarsus of *C. major* shows the same skeletochronology pattern. These data further confirm that the *C. major* bones found at the Bate cave belong to a single individual.

Conversely, the growth zones of the *C. ropalophorus* metatarsus (Fig. 8), the first that is 150 μm thick, while the other show the constant thickness of 70 μm. The estimated growth rate is 0.6 μm/d during the 2^nd^ year of life (corresponding to the first growth zone) and 0.3 μm/d from the 3^rd^ year of life onward. The absence of metaphyseal lines between the epiphyses and the diaphysis confirms that the individual was adult.

## DISCUSSION

### The diagnosis of pituitary gigantism: histomorphological and biomechanical considerations

**Figure 9:**
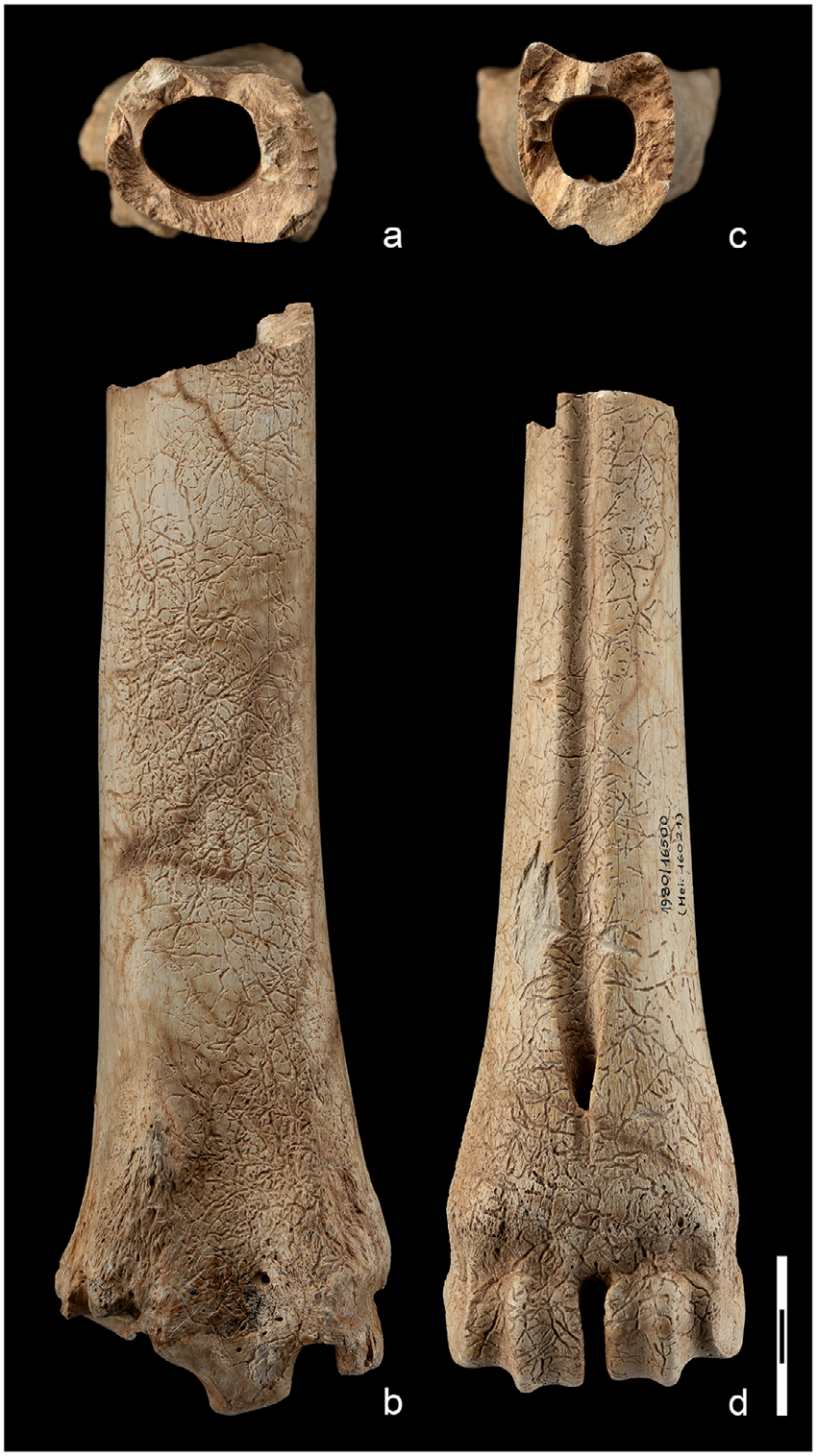
Tibia and metatarsus of *Eucladoceros giulii* from Untermassfeld (Germany): natural transversal section at about the mid‐point of the diaphysis (above); distal portions in frontal view (below). (a,b) Tibia dex. IQW 1980/16 501 (Mei. 16 022); (c,d) metatarsus dex. IQW 1980/16 500 (Mei. 16 021), barescale = 3 cm. Images credit Prof. Ralf‐Dietrich Kahlke (Senckenberg Research Station of Quaternary Palaeontology Weimar, Germany).

The results obtained from the macro–microscopical investigations of the paratype and holotype of the *Candiacervus major* species on the one hand show the presence of the plexiform, irregular Haversian, and dense Haversian bone tissue matching the typical bone histology of extant (Ruddle 1997; Gomez *et al*. 2013) and extinct deer species (Amson *et al*. 2015; Kolb *et al*. 2015b; Lyras *et al*. 2019). On the other hand, various anomalous peculiarities suggest that the Bate giant individual was affected by a generalized bone disorder. The main features highlighting this disorder are listed, discussed, and referenced in Table 6. The sum of evidence suggests a case of pituitary gigantism as the possible cause of the high stature and the peculiarity of long bone of *C. major*. We are aware that each single datum is not *per se* a proof, but all the results obtained together support this diagnosis as the most plausible. The main clinical evidence of a pituitary gigantism is the lengthening of long bones, associated with a reduction of the thickness of the diaphyseal bone wall (Mazziotti *et al*. 2018, 2019). The bone slenderness was the result of a fast, anomalous growing in length of the diaphysis due to the hyperstimulation of the growth plates located between each epiphysis and the diaphysis. Indeed, pituitary gigantism depends on GH excess that occurs during a young age when epiphyseal plates can excessively grow in length. This hormonal disorder can coexist during life with acromegaly, a phenomenon due to the same cause but which occurs in adulthood (Eugster & Pescovitz 1999; de Herder 2009; Melmed 2009). Moreover, the thinness of the diaphyseal bone wall in the long bones of the giant Cretan deer evidences a pathological condition, strengthening the diagnosis of a pituitary gigantism. Conversely, the diaphyseal bone walls of the small‐ and middle‐sized representatives of the genus *Candiacervus* are the thickest among those of the representatives of 24 genus of extinct and extant Cervidae (Amson & Kolb 2016). Amson and Kolb (2016) explained the peculiar thickness of the diaphyseal bone wall of the humerus, radius, and tibia of *C. ropalophorus* and *Candiacervus* spp. II as due to the phylogenetic affinity of these species with the giant deer of the Megacerini tribe (*Megaloceros* and *Sinomegaceros* sensu Vislobokova 2013). The value of *R*/*t* more than 3.0 both in tibia and in metatarsus of *C. major* is higher than those reported by Amson and Kolb (2016) ranging in the Cervidae the tibia from 1.5 *R*/*t* (*Puda puda)* to 2.9 (*Alces alces*), definitely higher than the *R*/*t* value of *C. ropalophorus* (1.53) and in *Candiacervus* spp. II (1.64), and higher even than the *R*/*t* in the tibia (1.98) metatarsus (2.07) of *E. giulii*, one of the continental deer more similar in size to *C. major* (Fig. 9 and Table 5).

**Table 6:** List of evidences at different observation levels supporting the diagnosis of pituitary gigantism

Observation level	List of evidences	Description	References
External morphology and morphometry	Extremely length of long bones	This is the main evidence suggesting the diagnosis of pituitary gigantism. It is due to an overstimulation of the chondroblasts present in the growth plates of long bones. Such condition is a typical effect of a great presence of growth hormone (GH)	Kronenberg (2003); Rossellò‐Diez & Joyner (2015); Tritos & Klibanski (2016)
	Slenderness index (SI = 0.066 in tibia and 0.074 in metatarsal bone)	The length of long bones is very disproportionate with respect to the transverse diameter of diaphysis	de Herder (2009); Melmed (2009)
	Articular surfaces very short	The size of the epiphyses of long bones do not follow the development of the diaphysis, so that the whole long bone appears disproportionate	Killinger *et al*. (2012)
	Bone fragility and fracture predisposition	The risk of fractures is very increased because the bones lose robustness and become fragile. The causes of this condition are attributable to an excess of GH that induces bone demineralization and bone reabsorption	Andreassen & Hoxlund (2001); Kužma *et al*. (2019)
Radiology	Extreme thinning of the diaphyseal bone wall (and consequently medullary cavity enlargement)	The thickness of the diaphyseal bone wall is the result of a process of enlargement of the medullary cavity due to reabsorption of subendosteal bone and a process of subperiosteal bone apposition. During the life, in physiological condition, these 2 processes are balanced and tend to gradually thicken the wall. In case of excess of GH, the enlargement of the medullary cavity prevails over the subperiosteal bone apposition, as confirmed by the growth zones delimited by LAGs	Eugster & Pescovitz (1999); Schmidt *et al*. (2007); Mazziotti *et al*. (2018, 2019)
	Bone radiotransparency	One of the effects of the GH on bones is a process of reabsorption of bone tissue stimulating the activity of osteoclasts. In case of overactivity of GH, as in case of pituitary gigantism, this process can be vigorous leading to a lowering of radiopacity typical of bone tissue (radiotransparency), and formation of bone pores within the compact bone tissue (bone porosity) which can give rise to large cavities hundreds of μm long	Ueland *et al*. (2002); Ueland (2005); Marotti *et al*. (2004); Dalle Carbonare *et al*. (2018)
	Bone porosity		
Histology	Presence of wide cavities in diaphyseal bone wall		
	Secondary osteons almost circular	Such information suggests that the bone tissue is subjected almost all to compression loads and not to mechanical stress in craniocaudal or mediolateral direction. It seems that the limb long bones were engaged in supporting the body weight than in participating in fast gaits	Zedda *et al*. (2015, 2019, 2020)
	Restricted growth zone between LAGs	This suggests an unbalance between the elongation of long bones and enlargement of their diaphysis, in favor of the former. This is the histological base of the bone slenderness	
Other indirect supports	Absence of carnivores predators	This condition allows the survival of individuals affected by pathologies restricting movements	Palombo & Zedda (2016); Lyras *et al*. (2019)
	Unique individual	The number of LAGs is the same in the tibia and metatarsal bone suggesting that both bones belonged to a 6‐ to 7‐year‐ old deer	

The thickness of the cortical diaphyseal bone is a strong genetic character so that it has been used as phylogenetic signal for evolutionary studies (Cubo *et al*. 2005; de Ricqlès *et al*. 2016; Mitchell *et al*. 2017). Therefore, the peculiar *R*/*t* value of *C. major* further supports the hypothesis of a pathological status of the Bate cave individual.

The bone reabsorption due to the overactivity of osteoclasts is another typical signal of pituitary gigantism (Andreassen & Hoxlund 2001). The reabsorption induces bone porosity in the epiphyses and the diaphysis causing the radiotransparency observed during radiological examination (Ueland *et al*. 2002; Ueland 2005; Dalle Carbonare *et al*. 2018). The bone radiotransparency of *C. major* (mean value 184) quantified by the colorimetric analysis is more than twice higher than that observed in the healthy bone tissue of *C. elaphus* (mean value 78).

The pathological process of bone remodeling and reabsorption causing radiotransparency was vigorous in the bone limbs of the giant Cretan deer, as documented by the presence of wide cavities in the diaphyseal bone. The cavities are very wide, reaching some hundreds of μm, and are definitely wider than the cavities (wide few tens of μm) present in herbivores during stress periods of their life such as pregnancy, lactation, or antler formation (Hillier & Bell 2007; Amson *et al*. 2015). Abnormal cavities inside the cortical diaphyseal bone, caused by an active osteoclastic bone resorption similar to those observed in *C. major*, have been described in a human metatarsus affected by segmental gigantism of upper and lower limbs (Marotti *et al*. 2004, p. 415, Fig. 4).

It might be speculated that the peculiar conformation of long bones may have made the large deer found at the Bate cave somehow vulnerable, affecting its life. It is challenging to say whether or not the propensity to fractures of long bones limited its movements to a fairly restricted range, hampering the chance to long distance movements de facto, potentially reducing the animal home range. However, the LAGs are very close to each other, indicating an extremely reduced subperiosteal bone formation activity. This means that the growth of the bones occurred almost all in length rather than in width. The growth rate of the subperiosteal diaphyseal bone in the giant deer (0.16 μm/d in the first 4 years of life and only 0.05 μm/d in the last 2 years before death) is lower than that estimated for *C. ropalophorus* by Kolb *et al*. (2015a), and in this study. Furthermore, since osteons showing an elliptical shape have been supposed typical of animal species with pronounced locomotor activity (e.g. mouflon compared to sheep (Giua *et al*. 2014), goat compared to sheep (Zedda *et al*. 2017), wild boar compared to domestic pigs (Zedda *et al*. 2019), and wild horse compared to domestic horse (Zedda *et al*. 2020)), the almost circular shape of the Bate individual osteons could suggest poor performance during fast and agile movements (Zedda *et al*. 2008, 2015; Zedda & Babosova 2021; Skedros *et al*. 2013), as already inferred by the morphological features shown by the autopodium bones (Caloi & Palombo 1995, 1996).

### The island rule

Following the observations on extant insular mammals by Foster (1964), the island rule describes the pattern of change in body size in insular vertebrates, predicting that large species tend to reduce their size while small species tend to increase their size (Van Valen 1973; Lomolino 1985, 2005; Lister 1989; Whittaker & Fernández‐Palacios 2007; Lokatis & Jeschke 2018). The generality of the island rule has been however questioned by various authors (see e.g. among others Meiri *et al*. 2004, 2006, 2008; Itescu *et al*. 2014). Differences in body size among the mainland ancestors and insular taxa, island geological history, time of isolation and insular ecosystem functioning may influence the speed and magnitude of body size changes (see e.g., Lomolino *et al*. 2012, 2013; Faurby & Svenning 2016). The processes related to ecological displacement may be a major force driving diversification in body size in the case of adaptive radiation (see e.g., Lomolino *et al*. 2013; Mazza *et al*. 2015; Palombo 2018). However, some departures from the predictions of this rule are recorded in extant large mammals getting larger than their mainland ancestor (e.g. the Kodiak bear *Ursus arctos middendorffi* and the Arctic fox *Alopex lagopus* from Mednyi Island [Bering Sea] (McNab 2001, 2010)) and in some small mammals getting smaller (e.g. *Mastomys huberti* (Ganem *et al*. 1995), *Sundamys muelleri* (Nor 1996), *Parantechinus apicalis* (Mills *et al*. 2004)).

The giant Cretan deer has been regarded for a long time as the most prominent departure from the island rule recorded among the fossil Pleistocene large mammals. The long bones of the giant deer from Bate cave, ascribed to *C. major*, are indeed longer than those of most of the roughly contemporaneous deer known on Greek mainland (cf. Capasso Barbato & Petronio 1986). The metatarsus, for instance, nearly reaches the size of *C. latifrons* metatarsal bones, is slightly larger than the largest known specimens of *Megaloceros giganteus* (length of the largest metatarsus = 391 mm) (Kazakhstan, Shpansky 2014), and is similar to the length of the largest specimens of extant *Alces alces* from Alaska (414.3 mm) (Silvia *et al*. 2014).

The oldest case of gigantism known to date in fossil large mammals is that of the Miocene cervoid *Hoplitomeryx* sp. from Scontrone (Abruzzi, central Italy) (Mazza *et al*. 2015). *Hoplitomeryx* sp. (consisting of few largely incomplete long bones and one astragalus larger but proportionally equivalent to the other astragali, Mazza *et al*. 2015, Fig. 4) is the largest among the 7 species of different sizes that lived contemporaneously on the palaeoisland. Mazza *et al*. (2015) explained its presence suggesting that the *Hoplitomeryx* “could freely develop new adaptive solutions, radiating from a stem species into a diverse set of niches and originating a set of coexisting species of different sizes, one of them giant” as already suggested for Cretan deer (Mazza *et al*. 2015, p. 276).

Consequently, the peculiar evolutionary radiation characterizing the Cretan deer could account for the unusually large size of *C. major*. Assuming as true that the ancestor of Cretan deer was a large or a medium‐size deer, the 4 species of size group 1 and 2 (*C. ropalophorous*, *C. devosi, C. listeri, C. reumeri*) (van der Geer 2018) significantly reduced their size (withers height about 50 cm), 2 (*C. cretensis* and *C. rethymnesis*) were moderately larger, while the stature of *C. dorothensis* falls in the range of modern red deer. In the Cretan predator‐free scenario, where the only other herbivore, *Palaeoloxodon creutzburgi*, could hardly be considered a competitor, these *Candiacervus* species differentiated each other in habitat and behavior, likely occupying, if available, all the niches typical of mainland deer.

The high stature and the poor limb maneuverability shown by *C. major* might be related to the advantage to avoid competition with other deer species in a low productivity environment by having access to a band of vegetation represented by sparse high trees, So that the gigantism hypothesis would be reasonable. Nevertheless, the amount of evidence resulting from this research highly questions the soundness of this hypothesis.

### Taxonomical and nomenclatural open issues

**Figure 10:**
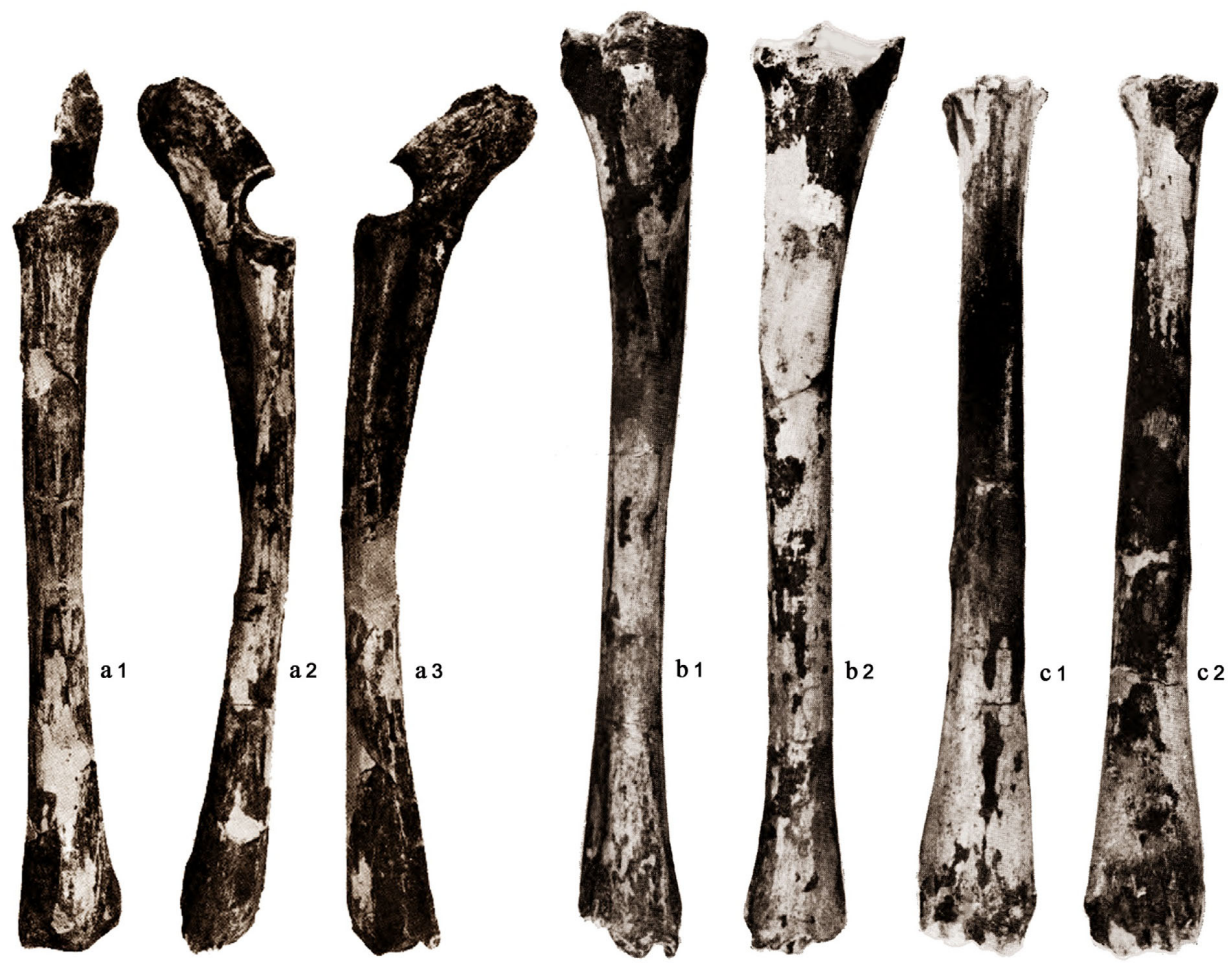
Holotype and paratypes of the species *Candiacervus major* (Bate cave, Rethymnon, Crete). (a) Radius and ulna (paratype) in anterior (a1), medial (a2), and lateral (a3) view; (b) tibia (paratype) in anterior (b1) and posterior (b2) view; c) metatarsus (holotype) in anterior (c1) and posterior (c2) view. Modified from Capasso Barbato and Petronio (1986, Tav I‐II).

The metatarsus and tibia herein described are, respectively, the holotype and the paratype (together with the radius‐ulna, which share with the other long bones the same elongated, slender shape) of *Candiacervus major* (Fig. 10), a species that is possibly represented by a single pathological individual at the Bate cave and by a couple of fragments at Liko. Therefore, a taxonomic and a nomenclatural review of large‐sized Cretan deer would be desirable in view of new diagnosis and selection of the types of the species. It seems rational to suppose that the “giant” deer affected by pituitary gigantism could be a pathological individual belonging to *C. dorothensis* that is the closest in size and the most similar as regards to long limb morphology and proportion, and this would imply that *C. major* is a subjective synonym of *C. dorothensis*. Further studies will clarify whether the giant deer from the Bate cave actually could be a pathological individual belonging to the *C. dorothensis* species or not. In both cases, a complex nomenclatural issue arises, whose discussion is beyond the purpose of this research.

### Concluding remarks

Macroscopical observations, osteometrical analyses, radiological and histological examinations of the tibia and metatarsus of the giant Cretan deer from the Bate cave support the hypothesis that the examined bones belong to an individual affected by pituitary gigantism. The results obtained deserve attention and further discussion since they describe for the first time a case of pituitary gigantism in an extinct mammal. The gigantism case presented here may significantly contribute to a better recognition of a syndrome rare in nature, poorly described in ancient remains and almost unknown in fossil mammals, and it could further validate the generality of the island rule. This highlights the potential that paleohistological investigations may have in paleontological studies to unravel paleoecological, evolutionary taxonomic, and nomenclatural issues.

## CONFLICT OF INTEREST STATEMENT

The authors declare no conflict of interest.
